# Intrathyroidal Relapse of Multiple Myeloma Mimicking Primary Thyroid Neoplasia: A Case Report and Literature Review

**DOI:** 10.1016/j.aed.2026.06.011

**Published:** 2026-07-01

**Authors:** Domenico Triggiani, Giuseppe Lisco, Giuseppina Renzulli, Mario Della Mura, Vincenzo Triggiani

**Affiliations:** 1 Interdisciplinary Department of Medicine, School of Medicine, University of Bari Aldo Moro, Bari, Italy; 2 Section of Molecular Pathology, Department of Precision and Regenerative Medicine and Ionian Area (DiMePRe-J), University of Bari Aldo Moro Bari, Italy

**Keywords:** case report, extramedullary myeloma, focal thyroid uptake, multiple myeloma, thyroid plasmacytoma

## Abstract

**Background:**

Thyroid nodules are highly prevalent in the general population, yet their diagnostic assessment may require accurate differential diagnosis in patients with hematologic malignancies. In this context, immune dysregulation, prior cytotoxic therapies, and the potential for atypical extramedullary involvement may confound conventional risk stratification algorithms. Although ultrasonography remains the first-line imaging modality, fine-needle aspiration with ancillary studies is often required for definitive characterization.

**Case report:**

A 47-year-old woman with relapsed multiple myeloma, previously treated with multiagent chemotherapy, radiotherapy, and autologous stem cell transplantation, was evaluated for biochemical and clinical disease progression. Bone marrow biopsy demonstrated no evidence of plasma cell infiltration. However, 18F-fluorodeoxyglucose positron emission tomography/computed tomography identified focal hypermetabolic uptake in the right thyroid lobe. Dedicated neck ultrasonography revealed a 26-mm solid, markedly hypoechoic nodule with peri-nodular vascularity and without definitive sonographic features of papillary thyroid cancer. Given the discordance between imaging findings and marrow status, ultrasound-guided fine-needle aspiration was performed. Cytologic evaluation demonstrated numerous atypical plasma cells, and immunocytochemistry confirmed lambda light chain restriction, consistent with clonal plasma cell proliferation. These findings established intrathyroidal involvement by multiple myeloma, representing an isolated extramedullary relapse in the absence of bone marrow disease.

**Discussion:**

Intrathyroidal manifestation of multiple myeloma is rare but clinically significant. In patients with systemic malignancies, fluorodeoxyglucose-avid thyroid nodules should prompt consideration of secondary involvement, even when bone marrow evaluation is negative. Conventional ultrasound-based risk stratification systems are primarily designed to detect epithelial thyroid malignancies and may fail to identify nonepithelial neoplasms. Cytologic assessment supplemented by immunophenotypic analysis remains essential for accurate diagnosis.

**Conclusion:**

This case highlights the importance of integrating clinical history, functional imaging, cytopathology, and immunocytochemistry to avoid diagnostic pitfalls and to guide appropriate oncologic management.

**Highlights**

• Incidental 18F-fluorodeoxyglucose-positron emission tomography/computed tomography (PET/CT) thyroid uptake discovered extramedullary multiple myeloma relapse: in a 47-year-old woman with relapsed multiple myeloma, PET/CT revealed focal right thyroid uptake amid skeletal progression. Ultrasound showed intermediate risk nodule. Thyroid cytology and immunohistochemistry confirmed atypical lambda-restricted plasma cells, confirming extramedullary plasmacytoma. PET/CT focal thyroid uptake can hinder ∼30% malignancy risk, vital for onco-hematologic surveillance
• Ultrasound classification systems have limitations in nonpapillary thyroid cancers: hallmarks validated for papillary carcinoma fail to detect medullary, oncocytic and undifferentiated carcinomas or secondary infiltrates. This nodule mimicked low-to-moderate suspicion for papillary thyroid carcinoma but concealed intrathyroidal myeloma. Perinodular vascularity without microcalcifications underscores need for adjunctive cytology in such a high-risk patient
• Thyroid cytology with immunocytochemistry differentiates clonal plasma cell disease: ultrasound-guided fine-needle aspiration and subsequent cytological and immunohistochemistry evaluations showed plasma cell morphology with lambda chain restriction. Enabled oncology referral for talquetamab, prioritizing proper pharmacological over surveillance or surgical management

**Clinical Relevance**

Positron emission tomography/computed tomography focal thyroid uptake in patients with multiple myeloma warrants fine-needle aspiration of avid nodules despite possible Thyroid Imaging Reporting and Data System constraints, with cytology and immunohistochemistry detecting rare extramedullary (intrathyroidal) relapse mimicking a low-to-moderate risk nodule and guiding systemic therapy for optimal management.

## Introduction

Thyroid nodules are highly prevalent in the general population, with ultrasound-detected nodules found in up to 68%, and a malignancy rate of around 5%.^1^^,^^2^ Ultrasonography remains the cornerstone of the initial evaluation, providing high sensitivity for nodule detection and characterization. However, classic sonographic hallmarks of malignancy – such as hypo-echogenicity, irregular margins, microcalcifications, increased vascularity, and a taller-than-wide shape – are primarily validated for papillary thyroid cancer.^3^ As a result, nodules lacking classic ultrasonographic features of malignancy may still conceal other malignant entities, including medullary thyroid carcinoma, follicular, oncocytic, and undifferentiated thyroid carcinoma, or nonthyroidal lesions.

In patients with onco-hematologic diseases, diagnostic challenges are amplified.^4^, ^5^, ^6^, ^7^ They frequently undergo complex treatments, such as chemotherapy, immunotherapy, or stem cell transplantation, all of which may alter immune response^8^ and endocrine homeostasis.^9^ These factors can influence both the presentation and management of thyroid nodules. Although primary thyroid lymphoma is rare, certain hematologic malignancies can localize to the thyroid, clinically, and radiologically mimicking thyroid nodules.

Since ultrasound imaging may yield atypical or inconclusive findings, 18F-2-fluoro-2-deoxyglucose positron emission tomography/computed tomography (18F-F-2-DG PET/CT) scan is increasingly utilized in this context.^10^ Focal areas of increased FDG uptake within the thyroid are uncommon but carry a high likelihood of malignancy, with reported rates up to 35% among those undergoing fine needle aspiration (FNA).^11^ The latter remains the definitive diagnostic assessment, providing essential cytological and immunophenotypic information that helps distinguish benign from malignant lesions and identify secondary involvement by hematologic or metastatic diseases.^12^ This information is crucial for therapeutic decision-making, allowing clinicians to differentiate nodules that require surgical intervention from those better managed with medical therapy.

Herein, we describe the diagnostic work-up of an incidentally detected intrathyroidal localization of multiple myeloma (MM) – an exceedingly rare manifestation of extramedullary disease – in a 47-year-old woman.

## Case Report

A 47-year female patient presented for endocrinology consultation to undergo FNA after an incidental discovery of thyroid nodule following a 18F-FDG PET/CT scan. Ten years before, she had been diagnosed with MM, initially presenting with a vertebral lesion at the thoracic site (T11) and treated with laminectomy followed by vertebral stabilization from T9 to T11. She received induction therapy with bortezomib, thalidomide, and dexamethasone regimen for 4 cycles, followed by autologous stem cell transplantation.

Three years later, she experienced disease relapse at the T11 site, for which she underwent radiotherapy and then second-line treatment with daratumumab, lenalidomide, and dexamethasone (DARA-Rd regimen), completing 22 cycles in 3 years. However – due to further skeletal disease progression – she underwent stabilization of the left femur and palliative radiotherapy, followed by third-line treatment with carfilzomib and dexamethasone (KD regimen) for 4 cycles and a second autologous stem cell transplantation. She continued with KD for 2 years, until a rib fracture following a sneeze occurred, leading the referring oncologists to investigate for disease progression. Following a 18F-FDG PET/CT scan, signs of additional skeletal involvement (bilateral iliac wings and right sided ribs 2-7) were found, as well as an unexpected focal uptake in the right thyroid lobe indicating the presence of a suspicious nodule. Concurrently, she underwent bone marrow biopsy which showed no evidence of plasma cell infiltration, and no monoclonal component was detected in serum or urine, indicating an exclusively extramedullary relapse pattern.

She was referred for endocrinological investigation to complete differential diagnosis. She had no family history of thyroid cancer, nor any personal history of childhood neck irradiation or prior neck surgery. Basic blood thyroid tests confirmed euthyroidism (thyroid-stimulating hormone, thyroid stimulating hormone 2 mIU/L; free thyroxine, fT4 1.2 ng/dL; free triiodothyronine, fT3 3.2 pg/mL), without evidence of thyroid autoimmunity (anti-thyroid peroxidase antibodies <34 IU/mL; antithyroglobulin antibodies <115 IU/mL). Neck ultrasound showed multiple nodules, with the largest located in the middle third of the right lobe, solid, primarily hypoechoic, with an inner well-defined hypo-anechoic area, lobulated margins, without echogenic foci, overall measuring 26 (antero-posterior, AP, diameter) × 14 (transverse, T, diameter) × 26 (longitudinal diameter) mm, and exhibiting peri-nodular vascular signals on Color Doppler (Fig. 1). Small reactive lymph nodes were also described in the left lateral cervical region. According to the European (EU)^13^ and American College of Radiology Thyroid Imaging Reporting and Data System,^14^ the nodule was classified as European Thyroid Imaging Reporting and Data System4 (intermediate risk) and TR4; a similar risk estimation was obtained using the American Thyroid Association risk stratification system (Table 1).^15^

**Fig. 1:**
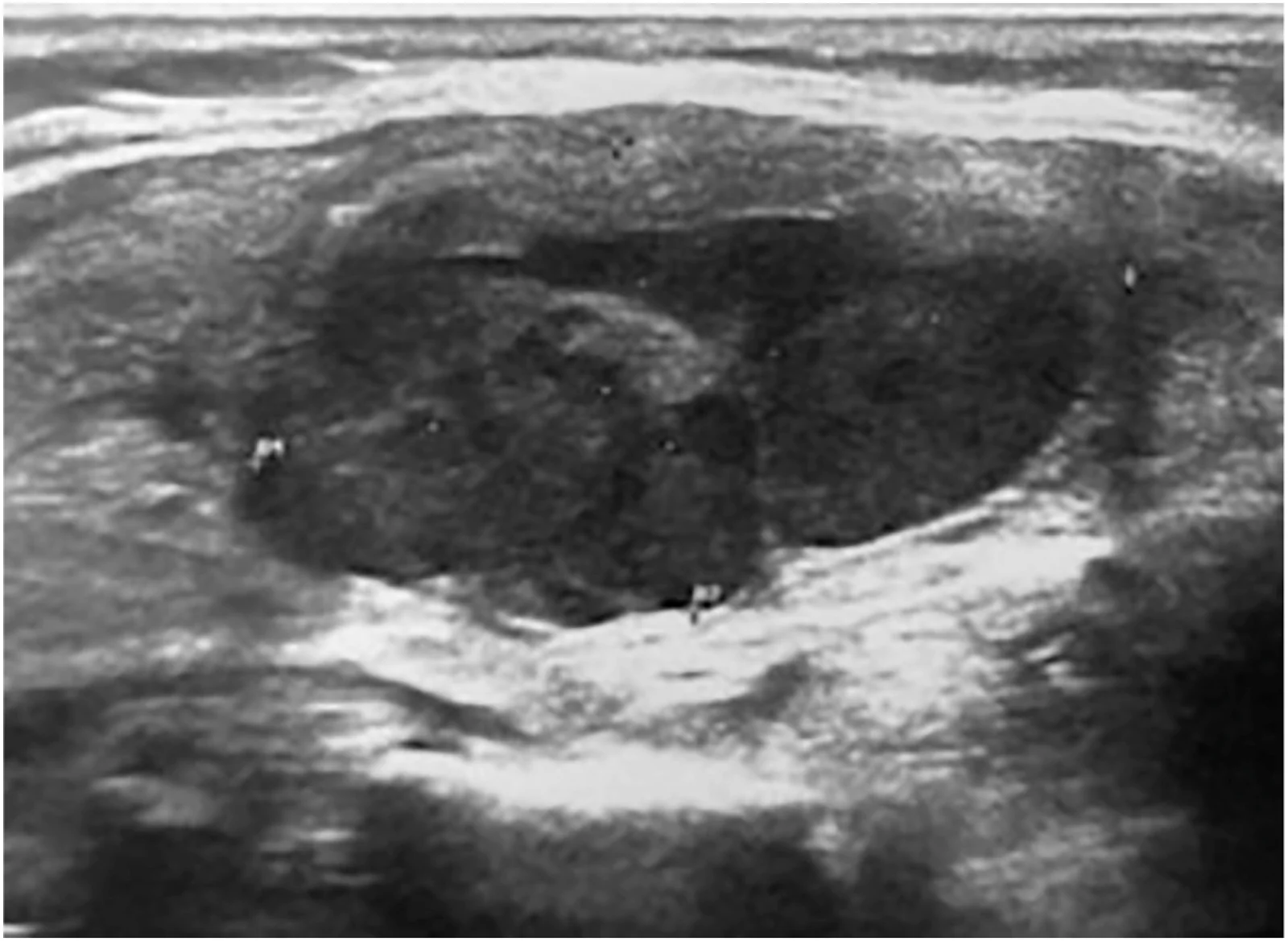
The nodule is located in the *middle* third of the *right* thyroid lobe, solid, predominantly hypoechoic, with a well-defined inner hypo-anechoic area, lobulated margins, echogenic foci absent, and peripheral vascular signals (not shown, B-mode freeze image). Overall dimensions: 26 mm (antero-posterior) × 14 mm (transverse) × 26 mm (longitudinal).

**Table 1:** Thyroid Nodule Classification Summary (Case: 26 × 14 × 26 mm; right lobe)

System	Key features driving score	Classification	FNA indication
EU-TIRADS^13^	Prevalently mild-hypoechoic	4 (Intermediate, 6-17% risk)	≥15 mm (met: FNA recommended)
	Oval shape (AP/T ratio 1.86, >1)		
	Lobulated margins (smooth)		
	No microcalcifications		
	Perinodular vascularity (type II, nonsuspicious)		
	Size: 26 mm		
ACR TI-RADS^14^	Composition: solid (2 pts)	TR-4 (Moderately suspicious)	≥15 mm (met: FNA recommended)
	Echogenicity: hypoechoic (2 pts)		
	Shape: wider-than-tall (0 pts)		
	Margins: lobulated (2 pts)		
	Echogenic foci: none (0 pts)		
	Total: 6 pts		
ATA^15^	Solid hypoechoic nodule	Intermediate suspicion pattern	≥10–15 mm (met: FNA recommended)
	Wider-than-tall shape		
	Lobulated but nonirregular margins		
	No microcalcifications		
	No clear high-risk features		
	Size: 26 mm		

Abbreviations: AP, antero-posterior diameter; ATA, America Thyroid Association; ATR-4, TI-RADS category 4; CR, American College of Radiology; EU-TIRADS, European Thyroid Imaging Reporting and Data System; FNA, fine-needle aspiration; T, transverse.

Table 1 summarizes the main findings of 2 of the most widely utilized thyroid nodule classification systems (EU-TIRADS, ACR-TIRADS, and ADA). Both flags intermediate suspicion based on hypoechogenicity + lobulated margins, but underestimate non-DTC malignancy (e.g., MM). PET avidity + onco-hematologic history mandated FNA override standard size thresholds and perfectly justifies clinical decision-making for this case.

Based on these findings, she underwent a standard FNA with multiple aspirations and cytological examination revealed numerous atypical plasma cells, with high proliferative index (Ki67 80%) and lambda light chain restriction, consistent with extramedullary localization of multiple myeloma (MM) (Fig. 2). The patient was therefore discharged and referred back to the oncology team to continue her diagnostic and therapeutic work-up in light of the confirmed extramedullary relapse of MM. She subsequently commenced fourth-line therapy with talquetamab, a fully humanized IgG4 bispecific antibody against cluster of differentiation 3 and GPRC5D.^16^

**Fig. 2:**
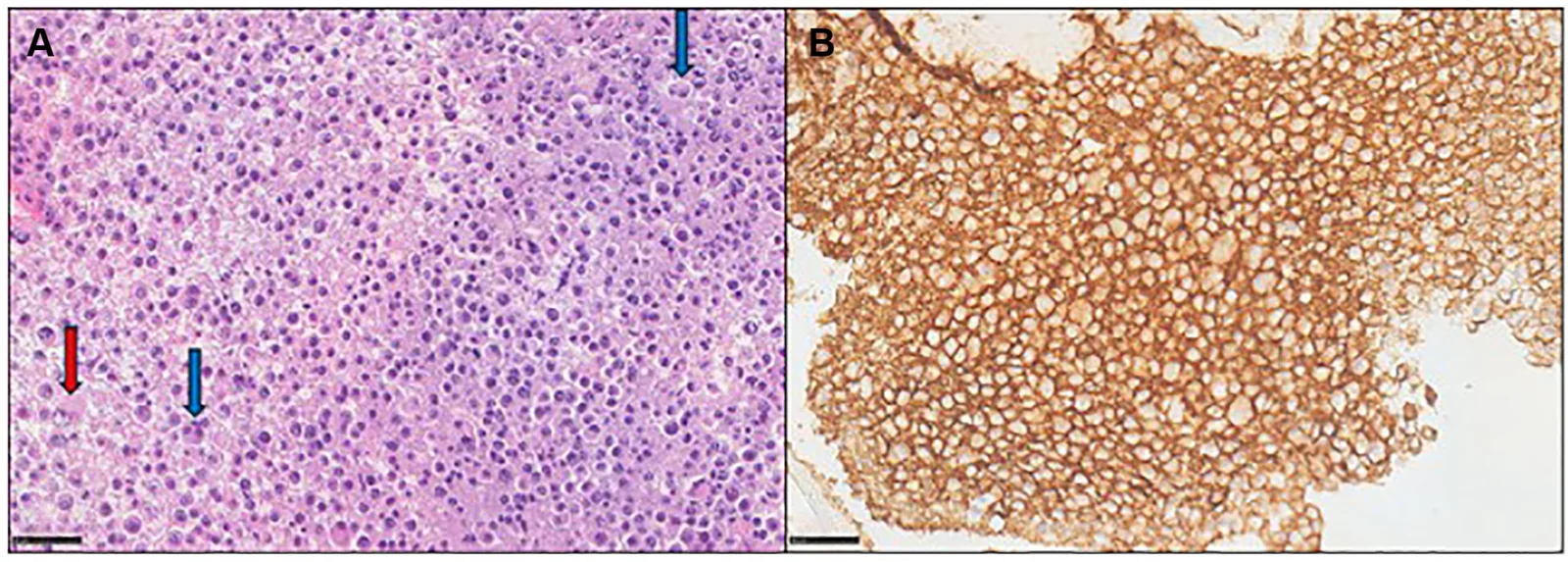
Histopathologic findings observed on the cell block obtained from fine-needle aspiration of the lesion: (A) cellular population composed of plasma cells, characterized by eccentrically located nuclei with cartwheel/clock-face chromatin and abundant eosinophilic cytoplasm; a certain degree of cellular atypia is evident, with occasional binucleated forms (blue arrows) and giant/pleomorphic cells (red arrow) (H&E, scale bar 50 μm); (B) immunocytochemical staining for CD138 demonstrates diffuse membranous positivity in the neoplastic cells, confirming their plasma cell lineage (IHC for CD138, scale bar 50 μm). These findings - along with the light-chain restriction - support the diagnosis of extramedullary localization of MM. CD, cluster of differentiation; IHC, immunohistochemistry; H&E, hematoxylin and eosin; MM, multiple myeloma.

## Methods

Thyroid nodule FNA was performed under real-time ultrasound guidance (Esaote MyLab 4) using a 22-gauge needle, with 2 dedicated passes for conventional cytology and one additional pass for ancillary studies on multiple samples. Smears were air-dried for May-Grünwald-Giemsa staining and alcohol-fixed for Papanicolaou staining.^17^ The cytologic material was also processed as a cell block to enable immunocytochemical characterization.^18^ The latter has been performed employing antibodies for cluster of differentiation 138 (plasma cell marker) and kappa and lambda light chains (to assess clonality). Cytologic interpretation focused on cellular morphology, degree of atypia, plasma-cell maturation, and the presence of light-chain restriction.

Cases were identified through a systematic literature search conducted on PubMed/MEDLINE and Google Scholar to identify relevant case reports.

## Discussion

MM is a clonal plasma cell dyscrasia characterized by the proliferation and accumulation of malignant plasma cells producing monoclonal immunoglobulins (M component), which typically accumulate in the serum and/or urine.^19^ The diagnosis relies on a combination of hematologic and imaging studies, including serum and urine protein electrophoresis with immunofixation, serum free light chain assay, and bone marrow aspiration and biopsy to grade plasma cell infiltration rate.^20^

In the diagnostic work-up, imaging modalities such as 18F-FDG PET/CT are crucial for detecting extramedullary lesions – especially in relapsed or refractory disease – providing essential information on the disease staging over time.^21^^,^^22^ Systemic involvement is primarily driven by bone marrow infiltration and the deposition of the M component in peripheral tissues. However, in some cases, extramedullary disease may arise due to the dissemination of plasma cells beyond the bone marrow microenvironment. The most frequently affected organs include the bones and kidneys, but virtually all tissues can be involved, such as skin, soft tissues, liver, lymph nodes, brain, breast, pleura, and pericardium.^23^ At that stage, the disease can progress aggressively even in case of optimal response of bone marrow involvement to therapy, as in the case herein described. This pattern is often attributed to clonal evolution and the emergence of plasma cell subpopulations with reduced dependence on the bone marrow niche, potentially driven by additional genetic mutations and altered expression of adhesion molecules and chemokine receptors.^24^

The head-neck region is among possible, though less common, sites of extramedullary localization of MM. Extramedullary plasmacytoma of the thyroid is itself an exceptionally rare entity; even more uncommon, however, is true intrathyroidal involvement by MM, with only few cases previously described (Table 2).^25^, ^26^, ^27^, ^28^, ^29^, ^30^, ^31^, ^32^, ^33^, ^34^, ^35^, ^36^, ^37^ The literature review by Refai et al^5^ reveals a male predominance (64%) and average age of presentation in the 7th to 8th decade. When present, thyroid involvement may clinically and ultrasonographically mimic thyroid cancer, creating significant diagnostic challenges.

**Table 2:** Review of Thyroid Plasmacytoma/Multiple Myeloma Cases Reported in Literature Since 1995

Author (y)	Sex	Age (y)	Presentation	FDG-PET	Diagnostic method	Outcome/Treatment
Alexander (2024)^25^	M	72	EMP thyroid + soft tissue/LN → MM	NR	Histology	Progressed to MM
Pasricha (2022)^26^	M	71	Rapidly enlarging FDG-avid neck mass mimicking ATC → thyroid plasmacytoma → MM	Yes	FNA + IHC	Systemic MM therapy
Sahu (2019)^27^	M	57	Thyroid nodule suspicious for carcinoma → plasmacytoma → MM	Yes	FNA	Led to MM diagnosis
Gochhait (2019)^28^	M	57	Plasmacytoma mimicking primary thyroid carcinoma	NR	Morphology	NS
Kashyap (2018)^29^	M	54	Thyroid cartilage/gland MM during work-up	Yes	PET/CT + biopsy	MM management
Singh (2017)^30^	M	57	FDG-avid thyroid nodule → plasmacytoma → later MM	Yes	FNAc	Developed MM
Park (2010)^31^	F	68	Disseminated plasmacytoma involving thyroid	NR	Histology	Disseminated disease
Özdemir (2009)^32^	M	57	Late light-chain MM relapse as thyroid EMP post-2nd ASCT	NR	Biopsy	Relapse management
Serefhanoglu (2008)^33^	F	76	Thyroid + pericardium EMP as MM presentation	NR	Biopsy	Initial MM therapy
Terrier (2006)^34^	F	78	Thyroid plasmacytoma	NR	Biopsy	CR to thalidomide/dex
Bourtsos (2000)^35^	M	73	Thyroid plasmacytoma mimicking MTC (amyloid pitfall)	NR	FNAc	NS
Schiller (1995)^36^	M	68	MM presenting as plasmablastic thyroid EMP	NR	Skeletal Rx + biopsy	Advanced disease
Vailati (1995)^37^	F	55	Cytologic thyroid plasmacytoma	NR	FNA	NS
Current Case	F	47	Intrathyroidal MM relapse (euthyroid nodule) post-2nd ASCT	Yes (incidental)	US-FNA + IHC (CD138+, λ-restricted, Ki67 80%)	Talquetamab (4th-line)
**Summary (*n* = 14)**	**64% M**	**Mean 65 ± 9**	**70% mimicking thyroid Ca; 50% →/known MM**	**∼50% (5/10 reported)**	**FNA/IHC 70%**	**50% systemic Rx; 20% CR**

Abbreviations: ASCT, autologous stem cell transplantation; ATC, anaplastic thyroid cancer; CD, cluster of differentiation; CR, complete response; EMP, extramedullary plasmacytoma; FDG-PET, 18F-fluorodeoxyglucose positron emission tomography; FNA, fine-needle aspiration; IHC, immunohistochemistry; LN, lymph node(s); MM, multiple myeloma; MTC, medullary thyroid cancer; NR, not reported; NS, not specified; Rx, treatment (therapy); US, ultrasound.

Table 2 comprehensively summarizes 14 reported cases of thyroid plasmacytoma or multiple myeloma (MM) involvement (1995 - 2024) - plus the current case - documenting consistent male predominance (64%), mean age 65 ± 9 y, frequent thyroid cancer mimicry (70%), FDG-PET avidity (∼50% of reported cases), FNA/IHC as primary diagnostics (70%), and variable outcomes (50% systemic therapy, 20% complete response, 50% MM progression/known disease). It underscores the rarity and diagnostic challenges, positioning the current incidental, euthyroid, Ki67-high relapse as representative yet distinct (younger female, post-dual ASCT).

In the case herein described, 18F-FDG PET/CT demonstrated a focal area of increased uptake within the thyroid gland. Neck ultrasound subsequently identified a nodule with features that – in a standard clinical context – would have raised moderate suspicion for DTC. However, the final decision to perform FNA was driven by the presence of focal thyroid uptake on PET/CT and the large nodule volume. Cytologic examination after multiple sampling with standard FNA revealed a dense infiltrate of atypical, proliferating plasma cells with marked light-chain restriction – an immunophenotypic hallmark of clonal plasma-cell proliferation – thereby confirming an extramedullary localization of MM rather than papillary thyroid malignancy. Focal thyroid uptake on PET/CT is uncommon, yet these nodules carry a remarkably high probability of representing nonbenign lesions, with malignancy rates approaching 30%.^38^ While many of these correspond to primary thyroid cancers, a subset reflects secondary involvement from extrathyroidal malignancies, including breast, colorectal, gastric, lung, melanoma, renal cell, ovarian carcinoma, and hematologic neoplasms such as lymphoma.^39^^,^^40^ In patients with known onco-hematologic disease, PET/CT may therefore serve as the first indicator of extramedullary spread, prompting targeted evaluation of sites that would otherwise appear incidental.

Cytology is crucial for diagnostic clarification,^41^ allowing reliable identification of plasma cells. This is achieved through careful morphologic assessment of the cellular elements – eccentrically located nuclei, coarse “clock-face” chromatin, a prominent perinuclear hof, and abundant basophilic cytoplasm – followed by immunocytochemical confirmation. Immunocytochemistry not only establishes plasma cell lineage – thereby excluding other lesions that can be composed of plasmacytoid-appearing cells (such as those medullary thyroid carcinoma) – but also allows assessment of κ or λ light chain restriction. The latter is crucial to differentiate reactive processes, characterized by a polytypic infiltrate, from neoplastic processes, in which the infiltrate is monotypic, as observed herein. The cytologic diagnosis is suggestive: while primary thyroid cancers typically require surgical management like thyroidectomy, extramedullary involvement by MM generally redirects therapy toward systemic hematologic treatment, with surgery or radiotherapy reserved for local control in select cases.

## Conclusion

The case highlights the increasingly valuable role of 18F-FDG PET/CT, together with the critical contribution of cytologic assessment, in the diagnostic work-up of nodular thyroid disease in patients with known malignancies, particularly hematologic neoplasms.

In such contexts, standard ultrasound-based risk stratification systems – primarily designed for identifying papillary thyroid cancer – may warrant a lower threshold for the evaluation of thyroid findings, while recognizing that the absolute risk of such extramedullary localization is low, as reflected by the limited number of cases reported in the literature. Clinicians should maintain a high index of suspicion when evaluating thyroid nodules in patients with a history of multiple myeloma or other systemic malignancies, especially in the presence of 18F-FDG PET/CT focal uptake. Integration of cytological, immunophenotypic, and imaging data are essential to guide appropriate oncologic management.

## Informed Consent

Written informed consent was obtained from the patient for publication of this case report and any accompanying clinical, laboratory, and imaging data. All procedures performed were in accordance with the international ethical standards and with the principles of the Declaration of Helsinki. Patient anonymity has been preserved.

## Funding

The authors received no financial support for the research, authorship, and/or publication of this paper.

## Disclosure

The authors have no conflicts of interest to disclose.
